# Mitochondrial genome maintenance—the kinetoplast story

**DOI:** 10.1093/femsre/fuac047

**Published:** 2022-11-30

**Authors:** Simona Amodeo, Irina Bregy, Torsten Ochsenreiter

**Affiliations:** Institute of Cell Biology, University of Bern, Baltzerstrasse 4, 3012 Bern, Switzerland; Graduate School for Cellular and Biomedical Sciences, University of Bern, Hochschulstrasse 6, 3012 Bern, Switzerland; Institute of Cell Biology, University of Bern, Baltzerstrasse 4, 3012 Bern, Switzerland; Graduate School for Cellular and Biomedical Sciences, University of Bern, Hochschulstrasse 6, 3012 Bern, Switzerland; Institute of Cell Biology, University of Bern, Baltzerstrasse 4, 3012 Bern, Switzerland

**Keywords:** kDNA, mitochondrial DNA, trypanosomes, mitochondrial DNA replication, kinetoplast

## Abstract

Mitochondrial DNA replication is an essential process in most eukaryotes. Similar to the diversity in mitochondrial genome size and organization in the different eukaryotic supergroups, there is considerable diversity in the replication process of the mitochondrial DNA. In this review, we summarize the current knowledge of mitochondrial DNA replication and the associated factors in trypanosomes with a focus on *Trypanosoma brucei*, and provide a new model of minicircle replication for this protozoan parasite. The model assumes the mitochondrial DNA (kinetoplast DNA, kDNA) of *T. brucei* to be loosely diploid in nature and the replication of the genome to occur at two replication centers at the opposing ends of the kDNA disc (also known as antipodal sites, APS). The new model is consistent with the localization of most replication factors and in contrast to the current model, it does not require the assumption of an unknown sorting and transport complex moving freshly replicated DNA to the APS. In combination with the previously proposed sexual stages of the parasite in the insect vector, the new model provides a mechanism for maintenance of the mitochondrial genetic diversity.

## Teaser

Summary of all currently known factors involved in kDNA replication in trypanosomes and a new model of the replication process.

## The mitochondrial DNA of trypanosomes and its unique structure

**Figure 1.**
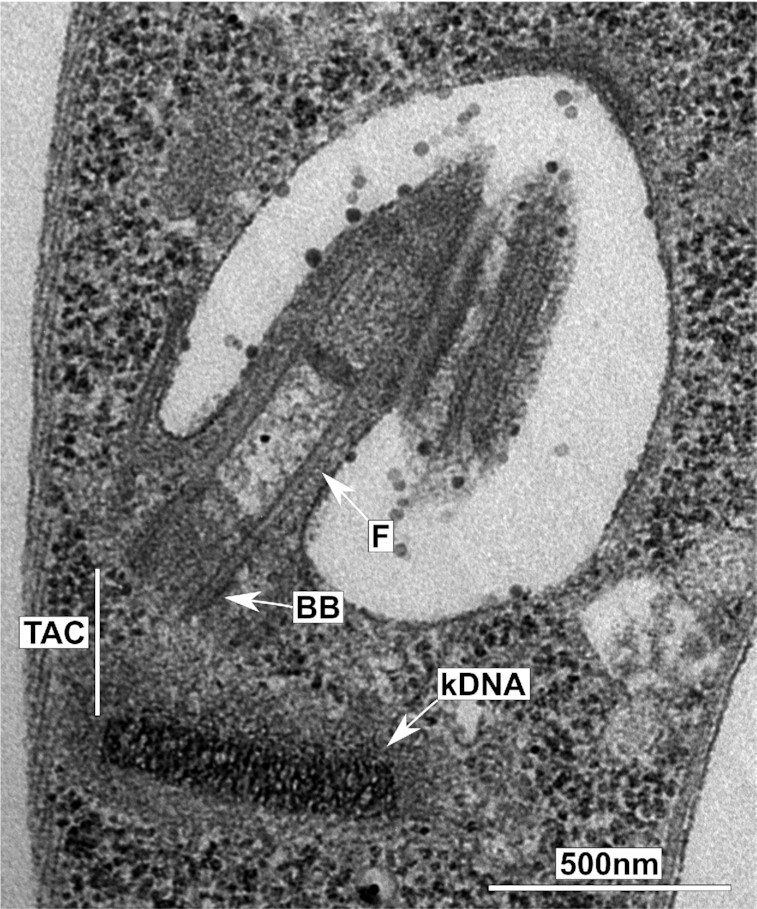
The kinetoplast of *T. brucei*. (A) Transmission electron microscopy image of a thin section through the basal body and kDNA network of *T. brucei*. In this orientation, the kDNA disc is cut orthogonal to its surface. BB, basal body; F, flagellum; TAC, tripartite attachment complex; and kDNA, mitochondrial genome.

In 1913, Robertson described a structure close to the base of the flagellum, which she named the kinetonucleus (Robertson 1913). In 1924, Bresslau and Scremin detected DNA within the kinetonucleus, which later was named the kinetoplast (Steinert et al. 1958, Burton and Dusanic 1968). In transmission electron microscopy, the kinetoplast of *Trypanosoma brucei* appears to be a disc-shaped, electron-dense structure within the cell’s single mitochondrion (Fig. 1). The biochemical isolation of kinetoplasts revealed that they consist of two types of DNA molecules, the maxi- and the minicircles. Minicircles in *T. brucei* are nonsupercoiled, 1 kb DNA molecules that are organized in an interlocked network. In *Crithidia fasciculata*, an insect parasite related to *T. brucei*, each minicircle is linked to approximately three other minicircles (Chen et al. 1995). The maxicircles are 23 kb in *T. brucei* and about 30 of these are interwoven into the minicircle network (Shapiro 1993, Chen et al. 1995, Cooper et al. 2019). Studies in *C. fasciculata* suggest that the electron-dense kinetoplast DNA (kDNA) structure is formed by sequential condensation events involving a number of kDNA associated, basic histone H1-like proteins (KAP; Xu et al. 1996, Yaffe et al. 2021). Overall, this results in a DNA structure with an estimated size of about 10^7^ kDa (Schneider 2001). *In situ*, the interlocked minicircles are stretched out and are oriented side by side, resulting in the typical striated network structure observed in transmission electron microscopy (Fig. 1). Prior to replication, the *T. brucei* kinetoplast disc is ∼450 nm in diameter and ∼150 nm in height (Jakob et al. 2016), the latter corresponding to approximately half the circumference of a minicircle (Shapiro and Englund 1995).

The minicircles contribute to over 90% to the mass of the network and each minicircle encodes three to five guide RNAs, which are required for maxicircle transcript editing (Hajduk and Ochsenreiter 2010, Aphasizhev and Aphasizheva 2011). A recent study in *T. brucei* has identified 391 distinct minicircle molecules that differ greatly in average copy number (Cooper et al. 2019). Despite the sequence diversity, the minicircles contain a conserved region of around 100–200 bp including the origin of replication (Chen and Donelson 1980). The leading-strand synthesis starts at the universal minicircle sequence (UMS; 12 bp), which is located within the conserved region (Ntambi and Englund 1985, Birkenmeyer et al. 1987). Also, the first Okazaki fragment is synthesized at an invariant hexamer site in the conserved region (Ray 1989). Furthermore, the minicircles also harbor a bent structure, which is caused by multiple A-tracts of 5 bp length that are positioned in phase with the helical repeat (Marini et al. 1982). The function of the helical bend region remains unclear but it has been hypothesized that it might be involved in the organization of the minicircles within the network (Shapiro and Englund 1995, Jensen and Englund 2012).

In *T. brucei ∼*30 identical maxicircles encode 18 proteins and two ribosomal RNAs (12S and 9S) but no tRNAs. The protein coding genes are mostly involved in the respiratory chain and include the ATP-synthase, cytochrome oxidase, NADH dehydrogenase subunits, and ribosomal components (ribosomal subunits uS12 and uS3m) as reviewed in (Feagin 2000, Schneider 2001). In addition, four open reading frames of unknown function are encoded. A total of 12 of the protein-encoding genes are cryptogenes and they require post-transcriptional modification through insertion/deletion RNA editing in order to be translatable. Specificity of the RNA editing process comes from the minicircle-encoded guide RNAs, that serve as templates for proper insertion and deletion (Hajduk and Ochsenreiter 2010, Aphasizheva et al. 2020).

**Figure 2.**
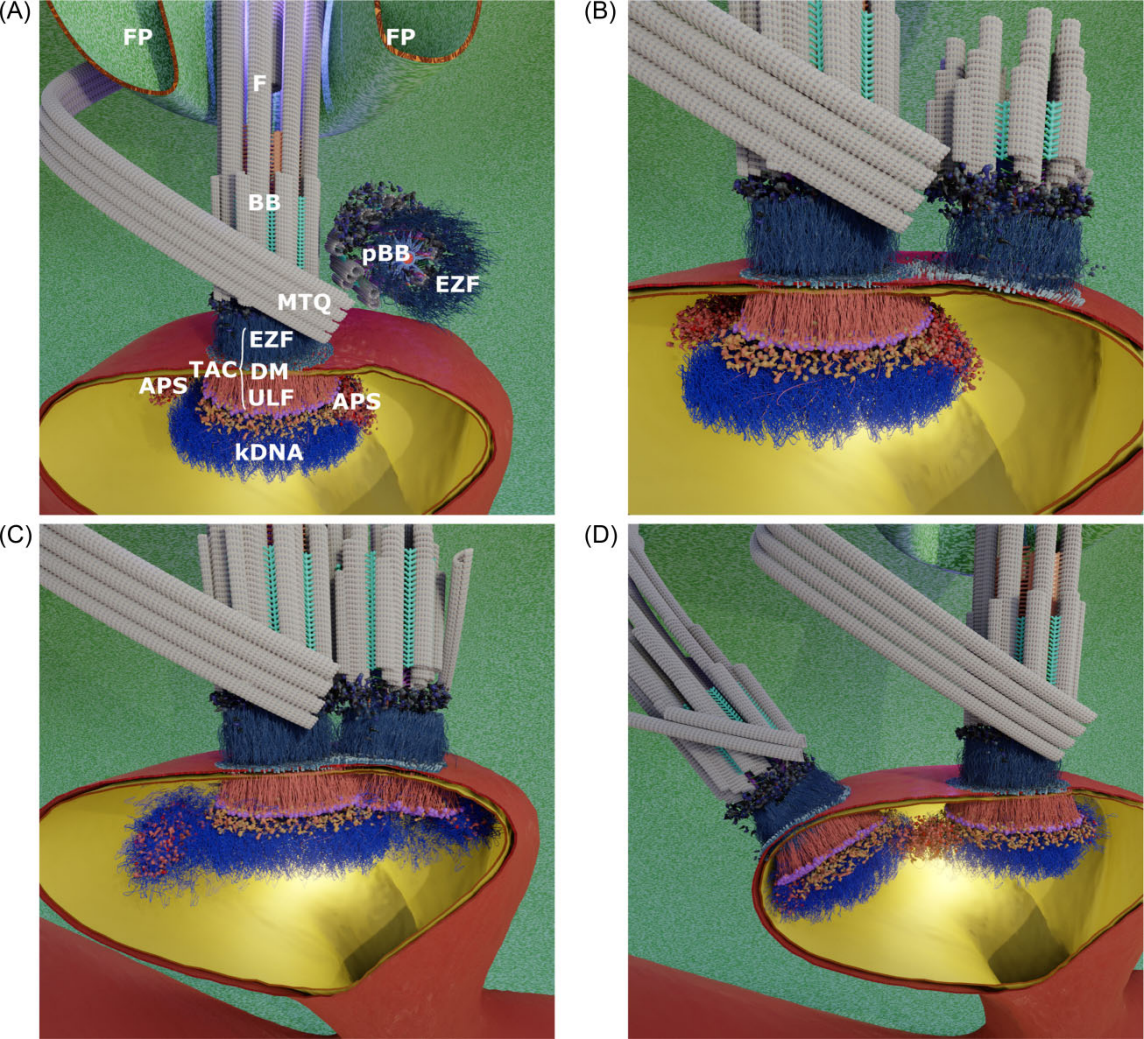
Overview of kDNA replication and segregation in *T. brucei*. **(A)** Dorsoventral view of the posterior region of a cell depicting the flagellar pocket, the basal bodies, and the mitochonrial membranes surrounding the kDNA at the beginning of kDNA replication. **(B)** and **(C)** Minicircles are released into the kinetoflagellar zone (KFZ), replicated, and reattached at the APS. Maxicircles are replicated, while remaining inside the growing kDNA network. Simultaneously to the kDNA replication process, the pro-basal body (pBB) tilts to face the mitochondrial membrane, and via the exclusion zone filaments (EZF), sets the base for growth of the new tripartite attachment complex (TAC). (C) and **(D)** During pBB maturation, the pBB rotates around the mature basal body (BB), and assembles the TAC proteins of the differentiated membranes (DM) and the unilateral filaments (ULF). (D) After completion of minicircle replication, the replicated kinetoplasts remain attached to each other by the maxicircles that accumulate between the two kDNA discs. Microtubule quartet (MTQ). The Images in this figure are stills from an animated model (see supplementary material).

Electron microscopy studies in combination with ethanolic phosphotungstic acid (E-PTA) have shown an asymmetric distribution of basic proteins around the kDNA disc with a strong enrichment at the two opposing regions that are known as the antipodal sites (APS; Fig. 2A; Melendy et al. 1988, Ferguson et al. 1992, Gluenz et al. 2007). It has been speculated that the very basic histone-like kDNA associated protein 4 (TbKAP4), TbKAP6, and the polymerase β-PAK could contribute to the E-PTA staining (Xu et al. 1996, Saxowsky et al. 2003, Gluenz et al. 2007, Wang et al. 2014). While *in vitro* experiments with isolated kDNA networks demonstrate that the histone-like basic proteins are able to compact or change the network structure, the precise localization of these proteins has not been shown (Xu et al. 1996, Wang et al. 2014). The APS are thought to contain free minicircle replication intermediates and 14 of the 19 characterized minicircle replication factors (Table 1; Melendy et al. 1988, Ferguson et al. 1992, Johnson and Englund 1998). Furthermore, the heterogeneous E-PTA staining within the APS suggests the presence of subdomains with different protein composition, which is supported by the differential localization of the mitochondrial topoisomerase II and ligase kβ in the APS (Downey et al. 2005, Gluenz et al. 2007). Although the APS have long been described, their composition, dimensions and dynamics remain poorly understood. In longitudinal kDNA sections, a thin rim of E-PTA staining further indicates the presence of basic proteins along the edge of the network disc (Gluenz et al. 2007). Such a confinement of the kDNA has been modeled as a potential driving force for minicircle network formation (Diao et al. 2012). However, similar to the APS, the identities of the basic proteins around the disc remain unknown.

**Table 1.** Localization of proteins associated with minicircle replication.

Protein name	Reported localization	Studied species^1^	Method	Consistent with current model^2^	Consistent with diploid model^3^	Reference
UMSBP1	KFZ	Tb	Cf antibody	Yes	Yes	Milman et al. (2007)
TOP2	APS*	Tb	Cf antibody	No	Yes	Wang et al. (2001)
RBP38	APS*	Tb	Ectopic, c-GFP	No	Yes	Liu et al. (2006)
PIF1	APS	Tb	*In situ*, 3xc-Myc	No	Yes	Liu et al. (2009a)
PIF5	APS	Tb	*In situ*, 3xc-Myc	No	Yes	Liu et al. (2009b)
PRI2	APS	Tb	*In situ*, c-HA	No	Yes	Hines and Ray (2011)
POLIB	KFZ	Tb	Antibody	Yes	No	Klingbeil et al. (2002)
POLIC	KFZ + APS	Tb	Antibody	Yes	Yes	Klingbeil et al. (2002)
POLID	KFZ + APS	Tb	*In situ*, c-PTP	Yes	Yes	Concepción-Acevedo et al. (2012)
p93	APS	Tb	Ectopic, c-3xHA	No	Yes	Li et al. (2007)
TOPIAmt	KFZ + APS	Tb	*In situ*, c-Myc	No	Yes	Scocca and Shapiro (2008)
SSE-1	APS	Cf	Ectopic, c-3xHA	Yes	Yes	Engel and Ray (1999)
Pol ß	APS	Tb	Ectopic, c-GFP	Yes	Yes	Saxowsky et al. (2003)
Pol ß-PAK	kDNA disc	Tb	antibody	Yes	Yes	Saxowsky et al. (2003)
LIG kα	kDNA disc	Tb	Ectopic, c-3xHA	Yes	Yes	Downey et al. (2005)
LIG kβ	APS	Tb	Ectopic, c-3xHA	Yes	Yes	Downey et al. (2005)
Polκ	APS	Tc	Ectopic, c-GFP	Yes	Yes	Rajão et al. (2009)
MiRF172	APS	Tb	*In situ*, c-PTP	Yes	Yes	Amodeo et al. (2018)

1 The proteins studied in species other than *T. brucei*, do have well-conserved homologs in *T. brucei*.

2 Minicircles are replicated in the KFZ. An unknown mechanism separates and transports replicated minicircles to the APS at opposing sides of the disc.

3 Minicircles replicate within the APS and are attached in that same area. This leaves both daughter minicircles generated from a single DNA molecule within the same side of the disc. It requires the mitochondrial genome to contain at least two copies of each minicircle.

* A star indicates inconsistency of earlier localization studies compared with the localization screen TrypTag. The localizations stated here, and used for further interpretations in this table, correspond to the findings of the single gene studies referenced in the respective row.

Kinetoplast biology, including replication and segregation of the kDNA, has been studied in a number of different Kinetoplastea, including *C. fasciculata, Leishmania tarentolae, Trypanosoma cruzi, T. evansi, T. equiperdum, T. gambiense, T. lewisi, T. mega*, and more recently, predominantly in *T. brucei*. In this review, we mostly focus on *T. brucei*.

## Replication of the kDNA

Replication of the kDNA begins prior to the nuclear S phase and leads to a gradual increase in size of the two kDNA lobes eventually forming two new kDNA discs (Fig. 2; Hoeijmakers and Weijers 1980, Guilbride and Englund 1998). In the current model, the minicircles are released from the network into the region between the kDNA disc and the mitochondrial membrane near the flagellar basal body (BB) called the kinetoflagellar zone (KFZ; Drew and Englund 2001). Here, they are replicated unidirectionally via theta intermediates (Englund 1979, Melendy et al. 1988, Sheline et al. 1989, Ryan and Englund 1989a,b, Abu-Elneel et al. 2001, Drew and Englund 2001). The two resulting daughter minicircles, which contain nicks and gaps from replication, are subsequently reattached to the network periphery at the APS in *T. brucei*. Only after reattachment to the network, the last remaining nicks and gaps are repaired, allowing the replication machinery to distinguish between replicated (nicked, gapped) and nonreplicated (covalently closed) minicircles. As generally accepted by the field, this mechanism is thought to ensure that each minicircle is replicated only once during each generation (Englund 1979, Pérez-Morga and Englund 1993a,b, Guilbride and Englund 1998, Liu et al. 2009a). The position where replicated minicircles are reattached to the kinetoplast network was shown to differ in *T. brucei* compared to other Kinetoplastea. While reattachment occurs at opposite or APS of the kinetoplast in *T. brucei*, the replicated circles are reattached to the network on the whole kinetoplast circumference in *C. fasciculata, L. tarantolae*, or*T. cruzi* (Guilbride and Englund 1998). Maxicircles, similar to the minicircles, replicate unidirectionally via theta intermediates. However, unlike the minicircles, they remain attached to the network during replication (Carpenter and Englund 1995). After replication the daughter networks are segregated, but initially remain connected by the “nabelschnur” that likely consists of maxicircles (Fig. 2D; Gluenz et al. 2011). The segregation of the kDNA is mediated by the physical connection of the kDNA to the basal bodies via the transmembrane spanning tripartite attachment complex (TAC; Ogbadoyi et al. 2003). The TAC and its components were recently reviewed and are not in the focus of this manuscript (Schneider and Ochsenreiter 2018).

## Minicircle replication—the known factors

In the following chapters, we discuss the properties of the proteins known to be involved in minicircle replication with a focus on components from *T. brucei*. We start with the release of minicircles into the KFZ, then move on to the replication and reattachment process. We have summarized the localization of each minicircle replication factor in Table 1. We, furthermore, provide a supplementary table that summarizes the current knowledge on all kDNA replication and segregation proteins including their localization and knockdown or overexpression phenotypes (Table S1, Supporting Information).

## Release of the minicircles for replication

### Universal minicircle binding proteins 1/2, UMSBP1, and UMSBP2 (Tb927.10.6070, Tb927.10.6060)

The conserved region of the minicircles contains the origin of replication and the UMS—the binding site of the UMS-binding protein (UMSBP) in *T. brucei* and *C. fasciculata*. In *C. fasciculata*, the protein localizes in the KFZ and is likely involved in replication initiation (Tzfati et al. 1992, Avrahami et al. 1995, Abu-Elneel et al. 1999, Milman et al. 2007). In *T. brucei*, two UMSBP orthologues containing zinc-finger domains have been identified (Milman et al. 2007, Klebanov-Akopyan et al. 2018). UMSBP2 localizes at the telomeres in the nucleus and is essential for their structure and function (Klebanov-Akopyan et al. 2018). Although it has not been directly demonstrated, UMSBP1 is likely the ortholog with functions related to kDNA replication. The simultaneous depletion of both UMSBPs in *T. brucei* results in nuclear mitosis defects, inhibition of minicircle replication initiation, and inhibition of BB segregation (Milman et al. 2007). This is well in line with the two separate localizations of the orthologues. It has been suggested that the mitochondrial UMSBP functions as an origin-binding protein, which triggers the initiation of replication through the recruitment of other replication factors (Milman et al. 2007). Furthermore, interaction of UMSBP with histone H1-like proteins and the ability of that protein complex to decondense kDNA networks have been shown. These decondensed networks then become accessible for topological decatenation by topoisomerase II, resulting in the release of minicircle monomers (Kapeller et al. 2011). Furthermore, UMSBP binding to the UMS induces conformational changes in the minicircles (Onn et al. 2006). Therefore, UMSBP1 may be involved in prereplication remodeling of the kDNA network by interacting with histone-like proteins, leading to accessibility of minicircles for replication enzymes.

### Mitochondrial topoisomerase II, TOP2 (Tb927.9.5590)

Based on the functional requirements, the most likely candidate for the release of minicircles from the kDNA network is TOP2. However, the depletion of TOP2 by RNAi does not lead to early defects in the release of minicircles and its localization is mostly at the APS, which is not compatible with the release of minicircles into the KFZ (Wang et al. 2000, Wang and Englund 2001, Downey et al. 2005, Kulikowicz and Shapiro 2006). The second function of TOP2 is the reattachment of minicircles to the network postreplication, which is supported by the functional studies that showed strong increase of nicked and gapped free minicircles upon RNAi depletion of the enzyme. Also, the localization of TOP2 to the APS supports its role in reattachment of minicircles (Wang and Englund 2001). Furthermore, TOP2 seems to be essential for the maintenance of the network structure, and therefore, might also be responsible for remodeling of the kDNA network during and after replication (Lindsay et al. 2008).

### Initiation of minicircle replication, synthesis

As described above, the minicircles replicate via theta intermediates. While the light strand synthesis is continuous and starts at the UMS (Englund et al. 1982, Ray 1989, Ferguson et al. 1992), heavy strand synthesis is discontinuous (Kitchin et al. 1984, Birkenmeyer and Ray 1986).

### Mitochondrial RNA-binding protein 38, RBP38 (Tb927.8.2740)

RBP38 contains a domain homologous to the antirestriction protein ArdC single-stranded DNA-binding domain, i.e. found in bacterial mobile elements (Krishnan et al. 2018). RBP38 binds the template strands of the UMS and the hexamer, and thus could be the minicircle replication origin recognition protein (Liu et al. 2006). How this potential function relates to UMSBP1 (that also binds to the UMS), remains to be investigated. One hypothesis is that UMSBP1 is required to recruit RBP38 to the UMS, which in turn allows binding of a helicase and a topoisomerase to unwind the DNA helix. Such an unwinding process could start at the origin of replication and proceed along the whole minicircle (Shlomai 2004, Liu et al. 2006). Surprisingly, RBP38 localizes at the APS and not in the KFZ, where initiation of replication is thought to take place (Liu et al. 2006).

### Mitochondrial helicases PIF1 and PIF5 (Tb927.11.6890, Tb927.8.3560)

Nomenclature of PIF helicases originates from their discovery in the yeast petite integration frequency locus (PIF; Boulé and Zakian 2006). *Trypanosoma brucei* encodes eight helicases related to the PIF1 helicase of yeast. A total of six of them are localized in the mitochondrion (PIF1, 2, 4, 5, 7, and 8) while PIF6 is found in the nucleus and PIF3 localizes throughout the cytoplasm (Liu et al. 2009a). A total of three of the PIF proteins are essential (PIF1, PIF2, and PIF8), and one (PIF5) shows a growth defect upon overexpression (Liu et al. 2009a,b). PIF1 and PIF5 function in minicircle replication. PIF1 localizes at the APS and its depletion leads to kDNA loss and accumulation of multiply interlocked, covalently closed minicircle dimers (termed fraction U), probably derived from late replication intermediates (Sundin and Varshavsky 1981, Liu et al. 2009b, 2010). The appearance of a fraction U type minicircle species was previously observed after depletion of TOP2 and POLIB by RNAi (Bruhn et al. 2010, Jensen and Englund 2012). PIF5, similar to PIF1, is localized to the APS, however, its depletion by RNAi does not show any detectable phenotype. Overexpression of PIF5, on the other hand, leads to reduced cell growth and a moderate loss of kDNA. Furthermore, PIF5 was shown to function in the process of primer removal in the minicircle Okazaki fragments (Liu et al. 2009b).

### Primase 2, PRI2 (Tb927.1.4010)

The *T. brucei* genome encodes two mitochondrial DNA primases named PRI1 and PRI2 (Hines and Ray 2010, 2011). Both enzymes have primase activity and are related to primases of eukaryotic viruses. PRI2 is a 129 kDa, very basic protein, i.e. conserved among the Kinetoplastea, except for its long N-terminal extension. RNAi-based depletion of the enzyme leads to a loss of kDNA with maxi- and minicircles being equally affected. Interestingly, RNAi also leads to an accumulation of covalently closed minicircles, and therefore, PRI2 seems to be directly involved in the initiation of minicircle replication. Whether the enzyme primes one or both strands at the replication origin, remains unknown (Hines and Ray 2011). Similar to the majority of the replication factors, PRI2 localizes at the APS, while initiation of replication is thought to take place in the KFZ (Hines and Ray 2011).

### Polymerases

In many of the well-studied eukaryotic model systems, the viral Pol γ is the replicative enzyme for the mitochondrial genome. A Pol γ homologue, however, is not encoded in the genome of *T. brucei* (Jensen and Englund 2012). Instead, the parasite uses seven DNA polymerases POLIA, IB, IC, ID, Polκ, Pol β, and β-PAK to replicate and repair the mitochondrial genome.

### POLIA, IB, IC, and ID (Tb927.4.2950, Tb927.11.4690, Tb927.7.3990, and Tb927.11.3260)

Three (POLIB, IC, and ID) of these four proteins that are related to the family A DNA polymerases are likely replicative enzymes, while POLIA is potentially involved in DNA repair (see under DNA repair; Klingbeil et al. 2002, Chandler et al. 2008).

### Polymerase IB, POLIB (Tb927.11.4690)

POLIB localizes to two sites in the KFZ, similar to UMSBP. Depletion of POLIB by RNAi leads to a decrease of nicked and gapped free minicircles, kDNA network shrinkage and eventually kDNA loss. This phenotype suggests a function in minicircle replication. The enzyme potentially functions as part of a heterodimeric replicase synthesizing leading as well as lagging strand of the minicircles (Klingbeil et al. 2002, Bruhn et al. 2010).

### Polymerase IC, POLIC (Tb927.7.3990)

In nonreplicating and early replicating kinetoplasts, POLIC localizes in the region of the KFZ, whereas in kinetoplasts that have been replicated, POLIC is predominantly found at the APS (Concepción-Acevedo et al. 2018). The differential localization is regulated by the N-terminal region of the protein (Miller et al. 2020). RNAi-based depletion of POLIC leads to network shrinkage with a faster loss of maxicircles than minicircles. Also, no loss of minicircle replication intermediates was observed, suggesting a possible role of POLIC in maxicircle replication (Klingbeil et al. 2002). Additionally, POLIC seems to also have a DNA polymerization independent role in the distribution of progeny kDNA networks (Miller et al. 2020).

### Polymerase ID, POLID (Tb927.11.3260)

POLID is generally distributed throughout the mitochondrion. In kinetoplast S phase, the protein is enriched at the kinetoplast disc and also at the APS (Klingbeil et al. 2002, Concepción-Acevedo et al. 2012). RNAi-based depletion of POLID leads to a rapid decline of mini- and maxicircles, eventually leading to kDNA loss. Furthermore, POLID depletion causes a transient accumulation of covalently closed as well as nicked and gapped minicircle replication intermediates just prior to kDNA loss. This behavior suggests a role in minicircle replication (Chandler et al. 2008). However, the decline of maxicircles during POLID RNAi is more rapid and complete than that of the minicircles, therefore, already Jensen and Englund suggested that the effect on minicircle replication might be indirect and that POLID is required for maxicircle replication (Chandler et al. 2008).

### Protein 93, p93 (Tb927.3.1180)

This 93 kDa, basic protein was initially identified in a screen for mitochondrially targeted proteins with differential, S phase-specific expression (Li et al. 2007). It localizes to the APS during the S phase of the cell cycle. Depletion of p93 by RNAi causes an early loss of nicked and gapped minicircle replication intermediates, suggesting its involvement in minicircle replication (Li et al. 2007). The protein is conserved in the Kinetoplastea, but its precise role in the replication process remains elusive.

### Mitochondrial topoisomerase IA, TOPIAmt (Tb927.10.1900)

The mitochondrial TOPIAmt of *T. brucei* is related to bacterial topoisomerases IA and reverse gyrases that can relax negatively supercoiled DNA (Scocca and Shapiro 2008). The protein displays a dynamic localization at the kDNA disc, mostly at the APS but also in the KFZ. It seems to play a role in the resolution of late theta structures (Scocca and Shapiro 2008).

## DNA repair

### Structure-specific endonuclease-1, SSE-1 (Tb927.10.340)

Structure-specific endonuclease-1 (SSE-1) was originally purified from *C. fasciculata* and was thought to be involved in minicircle primer removal (Engel and Ray 1998, 1999). More recently, SSE-1 was also studied in *T. brucei* (Liu et al. 2005). It localizes to the APS as it does in *C. fasciculata* (Engel and Ray 1999, Liu et al. 2005). SSE-1 RNAi depletion in *T. brucei* leads to an increase in nicked and gapped free minicircles and a delay in network segregation. This is likely caused by a defect or delay in gap repair, and thus supports its function in primer removal (Liu et al. 2005).

### Polymerase beta and polymerase beta-PAK, Pol Β and Pol Β-PAK (Tb927.5.2780, Tb927.5.2790)

Pol β and Pol β-PAK are the first examples of mitochondrial polymerase β enzymes (Saxowsky et al. 2002, 2003). Pol β-PAK was named after its N-terminal proline–alanine–lysine-rich (PAK) extension of about 300 amino acids. Both enzymes are thought to be responsible for gap filling and repair of minicircles albeit at different locations. Additionally, they show 5′-deoxyribose phosphate lyase activity suggesting a possible role in base excision repair (Saxowsky et al. 2002). Pol β is localized at the APS, where it is likely responsible for gap repair on the lagging strand prior to reattachment of the circles to the network. Pol β-PAK on the other hand, is found throughout the kDNA disc, where it is potentially required for gap repair prior to network segregation (Saxowsky et al. 2003). The different localization of the two enzymes is correlating with a difference in biochemical properties. While Pol β has its highest activity at pH 8.0, Pol β-PAK maximum activity is shifted towards a more basic pH (pH 9.0), potentially reflecting the environment within the kDNA disc.

### Mitochondrial DNA ligase homologs, LIG K-alpha and LIG K-beta (Tb927.7.610, Tb927.7.600)

Final gap repair and covalent closure of nicks of newly replicated minicircles occur after reattachment to the kDNA network and after all minicircles have been replicated. *Trypanosoma brucei* encodes two mitochondrial ligase genes. LIG kα and LIG kβ are highly divergent from other eukaryotic ligases (Downey et al. 2005). LIG kβ localizes at the APS and is thought to repair most of the gaps together with Pol β, before the newly replicated minicircles are reattached to the network. Interestingly, it does not seem to colocalize with TOP2, suggesting that distinct replication centers might exist. LIG kα localizes throughout the kDNA and is thought to repair the final gaps at the end of kDNA replication together with Pol β-PAK (Downey et al. 2005, Jensen and Englund 2012). Depletion of LIG kα leads to decrease in kDNA size followed by asymmetric division of the network and the associated complete loss of the kDNA.

### Polymerase kappa, Polκ (Tb927.11.8530)

This Polκ related enzyme was first discovered in *T. cruzi* and is conserved in most Kinetoplastea. In *T. cruzi*, Polκ localizes at the APS and at the BB proximal phase of the kDNA disc in the mitochondrion, which is very unusual when compared to orthologues from species outside the Kinetoplastea. *In vitro* studies show that Polκ efficiently bypasses 8-oxoguanine lesions, supporting its potential role in DNA repair (Rajão et al. 2009). Furthermore *in vivo* overexpression of the enzyme render the parasite less sensitive to gamma radiation suggesting a potential role of Polκ in homologous recombination (Rajão et al. 2009).

### Polymerase IA, POLIA (Tb927.4.2950)

POLIA is distributed throughout the mitochondrion and the depletion of POLIA does not lead to any detectable phenotype different from the wild-type cells. Together with phylogenetic relationship of its POLA domain to polymerase theta enzymes, which are mostly involved in DNA repair, a role in DNA repair rather than replication seems possible (Klingbeil et al. 2002).

## Reattachment of replicated minicircles at the APS

In most organisms, the RNA primers for the Okazaki fragments are immediately removed after replication. The current kDNA replication model suggests that after replication, the minicircles are moved from the KFZ to the APS and only there most primers are removed and the gaps between the Okazaki fragments are repaired (Ryan and Englund 1989a,b). Interestingly, at least one gap remains in the minicircles until after they are reattached to the growing disc. This last gap is repaired just prior to kDNA segregation and thus serves as a signal for the completion of replication.

### Minicircle replication factor 172, MiRF172 (Tb927.3.2050)

MiRF172 is a large, basic protein that localizes to the APS (Amodeo et al. 2018). RNAi-based depletion of MiRF172 leads to an initial increase in nicked, gapped minicircles that are not reattached to the network (Amodeo et al. 2018). Overall, this leads to a decrease in network size, i.e. seen in mini- and maxicircle abundance. While the precise function remains unclear, MiRF172 is possibly involved in the reattachment of replicated minicircles to the growing network. Interestingly, the protein was initially discovered in a screen for novel components of the kDNA segregation machinery (the TAC) but was then found to require both the kDNA itself and upstream components of the TAC for its localization and thus might provide a link between the two processes (Amodeo et al. 2018).

### Universal minicircle binding protein UMSBP1

UMSBP1 not only binds to the UMS, but also to the hexamer (the sequence at the start of the first Okazaki fragment; Abu-Elneel et al. 1999). While binding the template strand of the UMS, UMSBP also binds the complementary strand of the hexamer. The gap flanking the first Okazaki fragment is one of the last minicircle gaps being repaired after replication (Birkenmeyer et al. 1987, Ryan and Englund 1989a,b). This gap starts at the hexamer sequence and ends at the UMS. As suggested by Jensen and Englund, it is possible that UMSBP binds to the 5′ terminus of the Okazaki fragment region and protects it from premature repair (Jensen and Englund 2012).

## Maxicircle replication—the known factors

Little is known about the replication of maxicircles. Similar to the minicircles, they replicate unidirectionally via theta intermediates but remain attached to the kDNA disc at all times (Carpenter and Englund 1995). They form homo- and heterocatenates with each other and the minicircles, respectively. Just prior and at the beginning of the kDNA segregation process, at least some of the maxicircles are concentrated between the two separating discs and it is thought that they are eventually resolved through TOP2 activity.

### Mitochondrial primase 1, PRI1 (Tb927.8.2550)

RNAi against PRI1 causes loss of kDNA. The protein was localized predominantly to the APS, but a weak signal was also observed in the KFZ (Hines and Ray 2010). Although, the localization at the APS is not indicative of the involvement in maxicircle replication, it is thought that PRI1 is a maxicircle-specific replication protein. This is based on the observation that RNAi targeting PRI1 causes maxicircles to be depleted faster than minicircles. Furthermore, the depletion of PRI1 does not lead to loss of minicircle replication intermediates (Hines and Ray 2010).

### Mitochondrial helicase PIF2 (Tb927.11.6900)

PIF2 is a 115 kDa, basic protein localized to the KFZ in *T. brucei* (Table 1). The protein has ATP and Mg^2+^ dependent helicase activity and is one of the few known proteins to be clearly involved in maxicircle replication. RNAi targeting PIF2 leads to maxicircle loss, while overexpression of the protein causes a 3- to a 6-fold increase in maxicircle abundance (Liu et al. 2009a). Abundance of the protein seems to be directly or indirectly regulated by the HslVU protease (see below). Interestingly, despite the regulation by the HslVU protease, PIF2 abundance seems not to change during the cell cycle (Crozier et al. 2018).

### Polymerase IC and ID, POLIC and POLID (Tb927.7.3990 + Tb927.11.3260)

The knockdown phenotypes of POLIC and POLID suggest an involvement in maxicircle replication (see above). Both of these enzymes are likely to be replicative polymerases.

### Leucyl aminopeptidase metalloprotease 1, LAP1 (Tb927.8.3060)

LAP1 is an M17 family leucyl aminopeptidase metalloprotease (LAP) with a basic pI of 9.8. The protein shows a kinetoplast S phase-specific localization and it colocalizes with maxicircles at the nabelschnur during kDNA division, while it is also present at the APS. RNAi depletion of the peptidase leads to an increase of cells with two nuclei and two kinetoplasts that are arrested prior to cytokinesis. Overexpression on the other hand, results in loss of kDNA. While these experiments suggest a function of LAP1 in the kDNA segregation process, the details remain unknown (Peña-Diaz et al. 2017).

## Other kDNA-associated proteins

### Kinetoplast-associated protein 6, TbKAP6 (Tb927.10.8890)

TbKAP6 is an HMG-box containing protein, which is homologous to CfKAP4 in *C. fasciculata* and TcKAP6 in *T. cruzi* (Xu et al. 1996, Cavalcanti et al. 2009, Wang et al. 2014). It localizes throughout the kDNA disc during the entire cell cycle. Functional studies using RNAi targeting TbKAP6 show a decrease in minicircle release from the network prior to replication, eventually leading to loss of mini- and maxicircles and to partially disorganized kDNA networks (Wang et al. 2014). Overexpression on the other hand, leads to an increase in minicircle release, supporting the idea that the protein’s function might involve the release process prior to replication initiation (Wang et al. 2014). Recombinant versions of TbKAP6 were shown to promote topoisomerase II-driven release of minicircles from isolated *C. fasciculata* networks *in vitro* (Wang et al. 2014). Based on the experiments in *T. brucei, T. cruzi* and *C. fasciculata* it is likely that the function of TbKAP6 is involving the minicircle release process prior to replication.

### Tb927.2.6100

The highly basic protein (pI 10.7) encoded by the gene Tb927.2.6100 was identified by mass spectrometry of protein extracts from isolated kDNA. The protein seems to be restricted to *Trypanosoma* species and is not found, e.g. in *Leishmania* and *Crithidia*. It localizes at the kinetoplast and tandem affinity purification using a tagged version of Tb927.2.6100 identified mostly proteins of the mitochondrial ribosome and its assembly factors. Depletion of the protein by RNAi impairs growth, leading to small kDNAs, kDNA loss and decrease in guide RNAs and maxicircle transcripts. However, functional details of this protein remain unknown (Beck et al. 2013).

## Regulation and control of the kDNA replication

The *T. brucei* cell cycle can be divided into three to some extent independent subcycles (nuclear, kinetoplast, and cytoskeletal subcycle; Wheeler et al. 2019). However, the kinetoplast S phase is somehow timed in coordination with the nuclear S phase (Woodward and Gull 1990). Synthesis of kDNA initiates just before the start of the nuclear S phase and the replicated kDNA network divides just before mitosis. We do not know much about possible regulation mechanisms in the kinetoplast subcycle. Nonetheless, some discoveries about possible mechanisms regulating kDNA replication were made and are described in the following text.

### Mitochondrial helicase homolog PIF8 (Tb927.7.1000)

From the six mitochondrial helicases known, PIF8 is the smallest and most divergent among them. It likely does not have a helicase activity (Liu et al. 2009a, Wang et al. 2012). PIF8 mainly localizes at the phase of the kinetoplast, i.e. distal to the BB, but the localization pattern varies with different kDNA replication stages. Depletion of PIF8 led to moderate kDNA loss and only minor effects on covalently closed minicircles, while nicked and gapped minicircle replication intermediates decreased to around 50% compared to noninduced cells. Furthermore, depletion of PIF8 by RNAi leads to disorganization of the kDNA structure (Wang et al. 2012). Altogether this could suggests that PIF8 has a function in organizing parts of the replication machinery during the replication process.

### HslVU protease complex (Tb927.11.10240, Tb927.5.1520, and Tb927.11.12230)

HsIVU is a bacterial-like ATP-dependent protease complex consisting of three proteins in *T. brucei; o*ne HslV homologue (Tb927.11.10240) and two HslU homologues (Tb927.5.1520, TbHslU1 and Tb927.11.12230, TbHslU2; Li et al. 2008). In *T. brucei*, the three proteins are distributed throughout the mitochondrion with an increased concentration at the kDNA (Li et al. 2008). The protease complex is essential for *T. brucei* and depletion leads to a 20-fold increase in minicircles and a 3-fold increase in maxicircles. Additionally, the covalently closed, as well as nicked and gapped minicircle replication intermediates increase by 5- to 6-fold upon depletion of the enzyme complex. Furthermore, Hs1VU-depleted cells generate very large kDNA networks with a distorted structure. It has been suggested that HslVU is involved in controlling kDNA synthesis through the degradation of a positive regulator of DNA replication. Upon HslVU RNAi, this positive regulator may be stabilized, leading to kDNA over-replication and a significant increase in kDNA mass (Li et al. 2008). A target candidate for the trypanosome HslVU is potentially PIF2, as increased levels of PIF2 are detected upon HslVU knockdown (Li et al. 2008). Other targets of the *T. brucei* HslVU are currently unknown.

### Puf nine target 1, PNT1 (Tb927.11.6550)

PNT1 (Puf nine target 1) is a C11 cysteine peptidase, i.e. required exclusively for the maintenance of the kinetoplast (Grewal et al. 2016). RNAi targeting PNT1 leads to a loss of kDNA, while its overexpression causes the formation of extra kDNAs (called ancillary kDNAs), that are not connected to the basal bodies. PNT1 is localized at the opposing ends of the two growing kinetoplasts in the area of the APS. It is not clear how and whether PNT1 regulates kDNA replication, but it was shown that its activity is essential for parasite survival (Grewal et al. 2016).

### UMSBP—redox-regulated binding

Redox pathways have been shown to control biochemical processes such as transcription. Redox regulation was also observed for UMSBP in *C. fasciculata* and is the first example of a redox-regulated DNA synthesis protein (Onn et al. 2004, Shlomai 2010). The active form of UMSBP is fully reduced, more precisely, the cysteine residues of the zinc finger domains were observed to be reduced. Oxidation of the -SH to S-S renders the protein inactive, not binding to the UMS because zinc is no longer bound after oxidation (Onn et al. 2004). Further, NADPH stimulates the reduction of UMSBP and as a consequence leads to increased binding to the UMS (Sela et al. 2008, Shlomai 2010). The active and inactive form of UMSBP fluctuate in a cell-cycle-dependent manner in *C. fasciculata*. A total of two peaks of activity were observed during the kinetoplast S phase, and therefore, it was suggested that this might be a mechanism for kDNA replication regulation (Sela et al. 2008, Sela and Shlomai 2009).

## Division and segregation of the kDNA

The last step of the kinetoplast S phase is the division of the double-sized network. The kDNA is segregated by movements of the basal bodies that are attached to the kinetoplast through the TAC (Robinson and Gull 1991, Ogbadoyi et al. 2003). Simultaneously with the replication of the kDNA, the new flagellum starts to form. In the first step, the pro-BB’s proximal face tilts towards the mitochondrial membranes. Subsequently, the TAC is formed from the BB toward the kDNA (Fig. 2B and C). While the new BB is already attached to the kDNA, it rotates around the old BB, which is thought to help flagellar pocket biogenesis (Fig. 2C). It also possibly contributes to the bilobed shape of the replicated kinetoplasts during late S phase and beginning of segregation. After replication, the kinetoplasts remain connected to each other by the maxicircles, which become visible as nabelschnur when the distance between the segregating networks increases (Fig. 2D). With increasing distance between the daughter networks, the nabelschnur becomes longer and thinner and reaches at least 1 µm (Gluenz et al. 2007, 2011). The last step of kDNA division is the unlinking of the maxicircles by cleavage of the nabelschnur. As suggested by Jensen and Englund, this presumably is performed by TOP2 with some involvement of LAP1 (Jensen and Englund 2012, Peña-Diaz et al. 2017). After the division of the network, all the remaining nicks and gaps are repaired, resulting in two networks containing covalently closed DNA circles only.

## An updated model of mitochondrial genome replication in *T. brucei*—the loose-diploid model

The current model of kDNA replication suggests that the minicircles are released into the KFZ, and are replicated there. Then the two daughter minicircles are separated and migrate to opposite sides of the kDNA network, to be reattached at the APS. While this model elegantly explains the maintenance of two complete sets of minicircles after replication, the localization of several proteins involved in this process, as well as the localization of the DNA replication intermediates is not congruent with the model. For example, TOP2 is suggested to be responsible for the release of the minicircles into the KFZ but is localized mainly at the APS. Several proteins involved in replication initiation like RBP38, the helicases, and the primases, localize to the APS and not the KFZ and thus, their positioning is not consistent with the current model where the replication and segregation of the minicircles has to occur in the KFZ (Table 1). Also, especially the early replication intermediates are exclusively found in a region that could be the APS but not in the middle of the kDNA disc. Furthermore, the current model also requires the presence of a completely unknown sorting and transport mechanism that would ensure proper separation and movement of the two daughter minicircles to the APS post replication.

**Figure 3.**
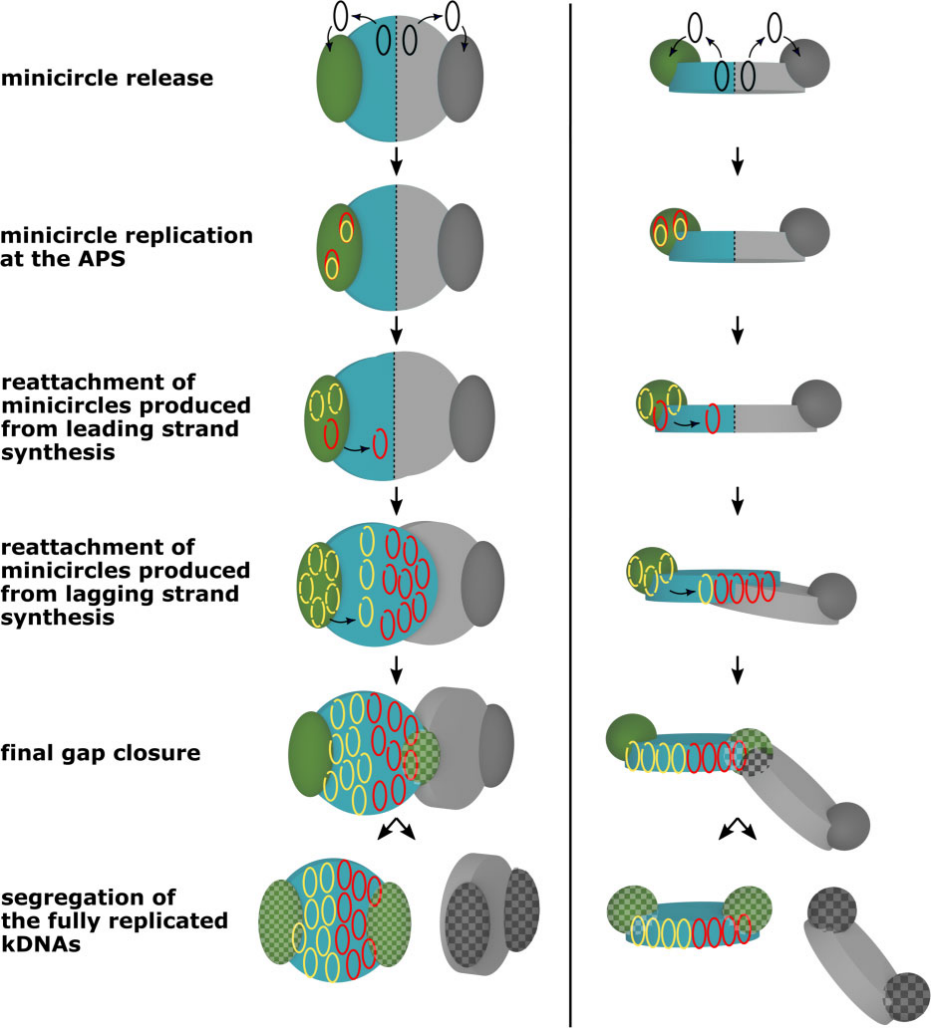
The diploid model of minicircle replication in the kDNA. Minicircles are released from the disc into the KFZ, bind to some replication factors, and move to the APS (green), where replication is initiated and proceeds via theta intermediates. Replication leaves the minicircle created from the leading strand with a single nick, i.e. rapidly repaired at the APS before the minicircle is reattached to the growing kDNA network. The lagging strand minicircle is left with multiple nicks and gaps to be repaired. Therefore, it will remain in the APS for a prolonged period of time (where the nicks and gaps are being repaired). As the disc is growing, the APS, and thus the site of minicircle reattachment, will move away from the position where the leading strand minicircle was reattached, leading to a spatial separation of the daughter minicircles. The depiction in this figure focuses on the processes in the disc on the left (depicted in cyan). The same process also applies to the disc growing on the right (depicted in gray). The APS of the disc in focus, are depicted in green, the network bound minicircles of the diploid genome are depicted in cyan; before DNA synthesis, free minicircles are shown in black; leading strand daughter circles are then depicted in red, while lagging strand daughter circles are shown in yellow. For simplicity we omitted the replication of the maxicircles in this figure.

Based on a comparison of the known minicircle replication factors and their localization we propose a new model (Table 1, Fig. 3, Supplementary movie). In such a model, the minicircles are released, replicated, and reattached at the same lobe of the disc (Fig. 3). To allow maintenance of the essential minicircle genome by this replication mechanism, the kDNA network must consist of a loose-diploid minicircle set (Fig. 3). Loose-diploid refers to the hypothesis that one set of essential minicircles is present, potentially in varying numbers, in each of the two lobes of the kinetoplast. We suggest that the entire minicircle replication occurs at the APS, where most of the replicative enzymes are localized. The APS themselves might not be static entities and might move along the kDNA disc or alternatively the kDNA itself might move relative to the APS during replication as it has been proposed previously in (Liu and Englund 2007). The mechanism of replication via theta intermediates and the removal of Okazaki fragments and gap repair has been elucidated and described in great detail (Ryan and Englund 1989a, Pérez-Morga and Englund 1993b). We assume the replication process is completed faster for the leading strand minicircles than for the lagging strand minicircles, since fewer gaps have to be mended in the leading strand. Consequently, the leading strand minicircle can be reattached shortly after replication, while gap repair for the lagging strand is still ongoing and would delay the reattachment of these minicircles. This leads to staggered distribution of the minicircles in the growing disc, allowing for an approximate separation between the two daughter minicircle sets, which is important for maintaining a bilobed minicircle distribution for the next generation. The loose-diploid model also provides an explanation for the observed dynamics in a population’s minicircle repertoire over time since it assumes a certain sloppiness in the redistribution of the minicircles in the disc (Savill and Higgs 1999, Cooper et al. 2019).

Maxicircles, similar to minicircles, are replicated unidirectionally via theta intermediates. They, however, remain attached to the disc and concentrate between the two replicated discs just prior to kDNA segregation (Carpenter and Englund 1995, Gluenz et al. 2011). The function of this positioning remains unclear. One could speculate that this is a consequence of the minicircle replication process, but it might also fulfill another function. The kDNA disc is rather compact and transcription of the maxicircle genes might be impacted by this. Thus, the “exposure” during kDNA segregation could also provide a window of opportunity for transcription to occur and would allow for the spatial separation of replication and transcription.

Interestingly, *T. brucei* is thought to produce gametes for sexual reproduction in the salivary glands of the tsetse fly (Peacock et al. 2011, 2014). Through the detection of a gamete specific protein (HAP2) numerous intermediate meiosis stages with varying kDNA/nucleus ratios have recently been discovered (Peacock et al. 2021). These cells even contained small kDNA networks that could be result of a meiotic segregation event. What remains unknown, is whether trypanosomes inherit their kDNA uni- or biparental (Peacock et al. 2011, 2014). The loose-diploid replication model introduced above would argue for a possible biparental inheritance of kDNA upon fusion of gametes. It is possible that the kDNA would undergo a “meiotic segregation” during gamete formation. This would subsequently allow fusion of the networks during gamete fusion to obtain a diploid minicircle set in the progeny. The diploid model in combination with biparental inheritance of kDNA would, thus provide a mechanism on how to maintain minicircle diversity during the life cycle of the parasite.

### Similarities and differences to the current model

The major differences to the current model of kDNA replication in *T. brucei* and *Crithidia* lie (i) in the assumption that the the kDNA disc of a cell prior to replication already contains two complete sets of the essential minicircles (is loose-diploid) and (ii) that each set is localized in the one lobe of the disc and (iii) thus the two minicircle sets are replicated separately.

Similar to the current models in *T. brucei* and *Crithidia*, the loose-diploid model also assumes (i) that the release of minicircles occurs laterally into the KFZ where they encounter the replication machinery and (ii) that lagging strand minicircle progenies require extensive gap repair and thus accumulate outside the disc, while the leading strand minicircle progenies are reattached immediately after replication is finished, which is similar to what has been described for the *Crithidia* minicircles (Kitchin et al. 1985). As a consequence, the lagging strand progenies are positioned differently in the growing disc (see Fig. 3). In addition to this passive process of daughter minicircle separation we also envision a more active mechanism involving either an actively moving replication or reattachment machinery or an oscillating and rotating kDNA disc as has been suggested previously for *T. brucei* and *Crithidia*, respectively (Liu and Englund 2007). Since there is no life cell imaging data following kDNA replication the actual dynamics of this process are difficult to evaluate.

### Where the loose-diploid model has shortcomings

The proposed model is based on the localization of mostly epitope tagged replication factors at the APS. Although tagging is a reliable technique in *T. brucei* we can not exclude that such proteins are mislocalized or non functional due to the epitope tag. However, at least for the topoisomerase TOP2 and several of the polymerases (PolIC, PolIB) mislocalization is unlikely since they were detected using antibodies targeting the endogenous protein. We assume that there are two separate replication machineries that only replicate the set of minicircles in their lobe of the kDNA disc. This likely requires some structural organization. Although there is no experimental evidence, the TAC could provide a structural element from which such a separation could be organized. The proper distribution of the minicircles in the kDNA lobe is key for the next generation. In addition to the passive mechanism of differential replication/repair (see above) we assume that either the reattachment machinery is moving along/across the disc or as proposed previously the kDNA disc itself is moving (Jensen and Englund 2012). Currently, we know of no mechanism explaining either of the movements.

In conclusion, we have summarized the current knowledge on 29 kDNA replication factors from *T. brucei* and *Crithidia* and suggest an updated model for *T. brucei* minicircle replication that does not require the assumption of a currently unknown sorting and transport complex moving freshly replicated minicircles to the opposing ends of the kDNA disc. Instead, it depends on a loosely diploid structure of the kDNA network and two replication centers at the APS. This is consistent with the majority of the localized replication factors (Table 1). In combination with the proposed sexual stages during the life cycle of the parasite, the new model would also provide a mechanism for maintenance of minicircle diversity. For future analyses of this replication model, it will be of great interest to assure wild-type-like expression levels of the tagged proteins of interest to decrease the risk of artifacts during detailed localization studies.

## Supplementary Material

fuac047_Supplemental_Files
